# Myrmarachnine jumping spiders of the new subtribe Levieina from Papua New Guinea (Araneae, Salticidae, Myrmarachnini)

**DOI:** 10.3897/zookeys.842.32970

**Published:** 2019-05-07

**Authors:** Wayne P. Maddison, Tamás Szűts

**Affiliations:** 1 Departments of Zoology and Botany and Beaty Biodiversity Museum, University of British Columbia, 6270 University Boulevard, Vancouver, British Columbia, V6T 1Z4, Canada University of British Columbia Vancouver Canada; 2 Department of Ecology, University of Veterinary Medicine Budapest, Budapest, H1077, Rottenbiller u. 50, Hungary University of Veterinary Medicine Budapest Budapest Hungary

**Keywords:** Ant mimicry, Astioida, molecular phylogeny, new genus, new species, Salticinae, Salticoida, taxonomy

## Abstract

A previously unreported radiation of myrmarachnine jumping spiders from New Guinea is described, which, although having few known species, is remarkably diverse in body forms. This clade is the new subtribe Levieina, represented by seven new species in three new genera. Within *Leviea***gen. n.** are three new species, *L.herberti***sp. n.**, *L.lornae***sp. n.**, and *L.francesae***sp. n.**, all of which are unusual among the myrmarachnines in appearing as typical salticids, not antlike. *Papuamyr***gen. n.** superficially resembles *Ligonipes* Karsch, 1878 or *Rhombonotus* L. Koch, 1879 as a compact antlike spider, but lacks their laterally-compressed palp and bears an ectal spur on the paturon of the chelicera. Two species of *Papuamyr***gen. n.** are described, *Papuamyromhifosga***sp. n.** and *P.pandora***sp. n.***Agorioides***gen. n.**, containing *A.cherubino***sp. n.** and *A.papagena***sp. n.**, is antlike, with the carapace sunken inwards (concave) between the posterior lateral and posterior median eyes. Phylogenetic analysis of data from the 28S, 16SND1, and COI gene regions of 29 species of myrmarachnines shows that the three new genera form a clade that is sister to the subtribe Myrmarachnina (*Myrmarachne* sensu lato), with the subtribe Ligonipedina less closely related.

## Introduction

The diverse and exquisitely antlike mymarachnine jumping spiders are found around the world, with hundreds of species in the Old World and a few in the Neotropics (Galiano 1969, Wanless 1978, Davies and Żabka 1989, Edmunds and Prószyński 2003, Ceccarelli and Crozier 2007, Edwards and Benjamin 2009, Yamasaki 2012, Yamasaki and Ahmad 2013, Yamasaki and Edwards 2013, Prószyński 2016, Pekár et al. 2017, World Spider Catalog 2018). Most belong to a group that we will treat as the subtribe Myrmarachnina (“*Myrmarachne* sensu lato”), consisting of *Myrmarachne* MacLeay, 1839, *Belippo* Simon, 1910, *Bocus* Peckham & Peckham, 1892 and several genera recently segregated from *Myrmarachne* (Prószyński 2016), all typically having forward-projecting male chelicerae and relatively delicate first legs. A smaller set of species falls into a cluster of genera with an embolus-bearing tegular groove, vertical chelicerae and enlarged first legs (including *Ligonipes* Karsch, 1878 and *Rhombonotus* L. Koch, 1879), which we treat as the subtribe Ligonipedina, and which forms the sister group to *Myrmarachne* sensu lato in recent molecular phylogenies (Bodner and Maddison 2012, Maddison 2016) and morphological phylogenies (Edwards and Benjamin 2009). The Ligonipedina is Australasian (Maddison 2015, World Spider Catalog 2018), as is the broader astioid diversification from which the myrmarachnines arose (Bodner and Maddison 2012), suggesting an original diversification of myrmarachnines in Australasia.

Accordingly, it is unsurprising that several distinctive new lineages of myrmarachnines have been found in New Guinea. We describe them here as three new genera, one of which (*Leviea* gen. n.) is unusual among myrmarachnines for being not in the least antlike. Even though the new levieines have body forms distinctive from other myrmarachnines, general body form is not necessarily a good clue to relationships in myrmarachnines because of their strong selective pressures for mimicry (Ceccarelli and Crozier 2007). Based only on body form, it would not be out of the question for the species described here to have been derived from within the Myrmarachnina. We therefore use molecular data to test the placement of our new species. The first molecular phylogeny of myrmarachnines with more than three species was that of Ceccarelli and Crozier (2007), who found two fairly distinctive clades among the Australian species of *Myrmarachne*. Subsequent molecular phylogenetic studies were done by Bodner and Maddison (2012), Merckx et al. (2015), Pekár et al. (2017), and Yamasaki et al. (2018), none fully incorporating data from the previous papers. We combine data from these studies to provide a sample of Myrmarachnina that is large enough to test whether the three new genera fall within that subtribe.

This paper began as a presentation in a symposium honouring Herbert W Levi in the 20^th^ International Congress of Arachnology (2016). Accordingly, it is dedicated to Levi and his service to arachnology. All of the specific epithets make reference to him and his family.

## Material and methods

The bulk of the material examined came from two expeditions to Papua New Guinea, one in 2008 (Maddison 2009, Maddison and Zhang 2011), the other in 2013 (Leponce et al. 2016), although a few specimens date back to 1968 (Balogh 1971). Preserved specimens were studied from these collections: Spencer Entomological Museum of the University of British Columbia (**UBC–SEM**, curator Wayne Maddison), Royal Belgian Institute of Natural Sciences (**RBINS**, curator Wouter Dekoninck), and Hungarian Natural History Museum (**HNHM**, curator László Dányi).

Specimens were examined under both dissecting microscopes (Nikon S800, Olympus SZ61) and compound microscopes (Nikon ME600L, Nikon Eclipse E200) with reflected light. Drawings were made with a drawing tube on a Nikon ME600L compound microscope.

Terminology is standard for Araneae. All measurements are given in millimeters. Carapace length was measured from the base of the anterior median eyes not including the lenses to the rear margin of the carapace medially; abdomen length to the end of the anal tubercle. The following abbreviations are used: **PLE**, posterior lateral eyes; **PME**, posterior median eyes (the “small eyes”); **RTA**, retrolateral tibial apophysis.

Taxa included in the molecular phylogeny are listed in Table 1, which also lists taxonomic authority for the species. The taxon sample includes one specimen of each of *Leviea*, *Agorioides*, and *Papuamyr*, other myrmarachnines, a set of astioid outgroups and a few more distant species from the Salticoida. The sequenced specimens of *Levieafrancesae* and *Agorioidespapagena* are the holotypes; that of *Papuamyromhifosga* is a paratype from the type locality. The *Ligonipes* specimen bears close resemblance to the male figured by Davies and Żabka (1989). The *Rhombonotus* specimen (from Australia, 26.235S, 152.640E) differs from the drawing in Davies and Żabka (1989) in having a slightly smaller spermophore loop. Except for the three new genera, most sequences come from already published works. It is not our purpose to resolve the phylogeny of *Myrmarachne* sensu lato, and so we include a small sample of 24 species, diverse enough to test whether the levieines are inside or outside the group. We include most *Myrmarachne* sensu lato used by Maddison (2016), and a selection of species from sequences obtained by Ceccarelli and Crozier (2007) (with identifications from Ceccarelli 2010), Jang and Hwang (2011), Merckx et al. (2015), Pekár et al. (2017), and Yamasaki et al. (2018). From the published sequences we excluded specimens known only from COI because of its poor phylogenetic performance when analyzed alone (Hedin and Maddison 2001, Maddison et al. 2014). We also excluded duplicates of species and most lacking an identification to species or species group. The only new data from *Myrmarachne* sensu lato are from a Neotropical species (M.cf.mocambensis from Ecuador, 2.9962S, 78.4558W), *Emertoniusmalayanus* (from Malaysia, 4.04N, 114.816E), and *Myrmarachnecornuta* (from Malaysia, 4.0432N, 114.8110E).

New DNA sequences of the genes or gene regions 28S, 16SND1, and COI were obtained using the protocols of Zhang and Maddison (2013) and Maddison et al. (2014). The first of these genes is nuclear; the last two are mitochondrial. Prior to phylogenetic analysis, multiple sequence alignment was done for 28S and the noncoding portion of 16SND1 with MAFFT v7.407 (Katoh et al. 2002, 2005) using the LINSI option (--localpair --maxiterate 1000), run via Mesquite (version 3.51, Maddison and Maddison 2018b). Maximum likelihood phylogenetic analyses were run using IQ–TREE version 1.6.7.1 (Nguyen et al. 2015), run via the Zephyr package (version 2.11, Maddison and Maddison 2018a) of Mesquite. The data were partitioned for most analyses, allowing the possibility of separate rates and substitution models. Initial partitions were 28S, 16S, ND1 position 1, ND1 pos. 2, ND1 pos. 3, COI pos. 1, COI pos. 2, COI pos. 3. Because the options “-m MFP -spp” were used, IQ–TREE inferred models of evolution for each partition (Kalyaanamoorthy et al. 2017), and whether to merge partitions (Chernomor et al. 2016). We ran 100 separate search replicates for the maximum likelihood tree for the concatenated analysis and for each gene separately. We performed a standard bootstrap analysis with 1000 replicates and the same model and partition settings.

Sequences obtained are deposited in GenBank (Table 1). Alignments and trees are deposited in the Dryad data repository (http://doi.org/10.5061/dryad.c2c0p0v).

**Table 1.** Specimens used for molecular phylogeny, with Genbank accession codes (*=previously published). Second column lists source publication as Citation:Voucher specimen, with citation codes: *BM2012*: Bodner and Maddison 2012; *CC2007*: Ceccarelli and Crozier 2007; *JH2011*: Jang and Hwang 2011; *M2016*: Maddison 2016; *M+2008*: Maddison et al. 2008; *M+2015*: Merckx et al. 2015; *M+2014*: Maddison et al. 2014; *MH2003*: Maddison and Hedin 2003; *P+2017*: Pekár et al. 2017; *Y+2018*: Yamasaki et al. 2018; *ZM2013*: Zhang and Maddison 2013. Specimens newly sequenced in bold.

	Citation: voucher specimen	28S	16SND1	CO1
**Non-astioid outgroups**				
*Afromarengo* sp.	BM2012:MRB262	JX145758*	JX145905*	JX145682*
Baviaaff.aericeps Simon, 1877	M+2008,M+2014:d079	EU815490*	KM032925*	EU815603*
*Dendryphanteshastatus* (Clerck, 1757)	M+2007,M+2014:d043	EF201646*	KM032927*	KM033228*
*Evarchaproszynskii* Marusik & Logunov, 1998	MH2003:S232; BM2012:d096	DQ665765*	DQ665723*	AY297379*
Idastrandiacf.orientalis (Szombathy, 1915)	M+2008:d108	EU815535/ EU815496*	EU815560*	EU815608*
*Omoedusorbiculatus* (Keyserling, 1881)	BM2012:JXZ136; ZM2013:JXZ088	JX145762*	KC616047*	KC615792*
**Astioid outgroups**				
*Apriciajovialis* (L. Koch, 1879)	M+2008:d021	EU815472*	EU815544*	EU815588*
*Arasiamollicoma* (L. Koch, 1880)	M+2008:d046	EU815483*	EU815550*	EU815598*
*Helpisminitabunda* (L. Koch, 1880)	MH2003:S194/S195; M+2014:d265	AY297282*	AY296700*/AY297345*	KM033227*
*Heratemitaalboplagiata* (Simon, 1899)	MH2003:S266	AF327934*	AF327962*/AF328021*	AF327991*
*Neonnelli* Peckham & Peckham, 1888	MH2003:S310	AF327931*	AF327959*/AF328018*	AF327988*
*Nungiaepigynalis* Żabka, 1985	M+2014:d221	KM033192*	KM032924*	
*Orthrusbicolor* Simon, 1900	MH2003:S192	AY297286*	AY296704*/AY297349*	AY297413*
*Papuaneontualapa* Maddison, 2016	M2016:d302/JXZ267	KY200845*	KY200842*	
*Penionomus* sp. [New Caledonia]	M+2008:d122	EU815498*	EU815561*	EU815610*
*Sandalodesbipenicillatus* (Keyserling, 1882)	M+2008:d019	EU815471*		EU815587*
*Triteplaniceps* Simon, 1899	MH2003:S197	AY297290*	AY296708*/AY297353*	AY297417*
*Viciriapraemandibularis* (Hasselt, 1893)	BM2012:d183	JX145757*	JX145904*	
** Ligonipedina **				
*Ligonipes* sp. 1 [Australia]	M+2008:d048	EU815484*	EU815551*	EU815599*
Rhombonotuscf.gracilis L. Koch, 1879 [Australia]	This:**GLR16-26**	MK716310	MK716304	
** Levieina **				
*Agorioidespapagena* sp. n.	This:**d253**	MK716311	MK716305	
*Levieafrancesae* sp. n.	This:**d254**	MK716312	MK716306	
*Papuamyromhifosga* sp. n.	This:**d267**	MK716313		
** Myrmarachnina **				
Belippocf.ibadan Wanless, 1978	BM2012,M2016:MRB118	JX145748*	KY200840*	JX145674*
*Emertoniusmalayanus* Edmunds & Prószyński, 2003	Y+2018:TYMLY02; This:**SWK12-1851**	MK716314	MK716307	LC193966*
Myrmageaff.gedongensis (Badcock, 1918)	BM2012:MRB117	JX145750*	JX145899*	JX145676*
Myrmapanacf.mocamboensis (Galiano, 1974)	This:**MRB152**	MK716315	MK716308	
*Myrmaplataplataleoides* (O. P.-Cambridge, 1869)	BM2012:MRB114	JX145754*	JX145902*	JX145680*
*Myrmarachneassimilis* Banks, 1930	MH2003:S149	AY297284*	AY296702*/AY297347*	AY297412*
*Myrmarachneaurea* Ceccarelli, 2010	CC2007: sp. B type 1		DQ373010*	DQ372996*
*Myrmarachnebicolor* (L. Koch, 1879)	P+2017:Myrm15		KT364840*	
*Myrmarachnecornuta* Badcock, 1918	M+2015:PK373; This:**SWK12-3302**	MK716316	MK716309	KP978509*
*Myrmarachneerythrocephala* (L. Koch, 1879)	P+2017:Myrm33		KT364853*	KT364810*
*Myrmarachneevidens* Roewer, 1965	BM2012:MRB249	JX145752*		JX145678*
*Myrmarachnefoenisex* Simon, 1910	BM2012:MRB254	JX145753*	JX145901*	JX145679*
*Myrmarachnegurgulla* Ceccarelli, 2010	CC2007: sp. D type 1		DQ373013*	DQ372994*
*Myrmarachnejaponica* (Karsch, 1879)	JH2011:LEGO_44_48	JN817063*	JN816647*	JN817283*
*Myrmarachneluctuosa* (L. Koch, 1879)	P+2017:Myrm24		KT364846*	KT364806*
*Myrmarachnemacleayana* (Bradley, 1876)	P+2017:Myrm1		KT364827*	
*Myrmarachnerubra* Ceccarelli, 2010	CC2007: sp. A type 3		DQ373015*	DQ372999*
*Myrmarachnesmaragdina* Ceccarelli, 2010	P+2017:Myrm10		KT364835*	KT364797*
*Myrmarachnestriatipesstriatipes* (L. Koch, 1879)	P+2017:Myrm31		KT364851*	
*Myrmarachne* sp. (*tristis* group) [South Africa]	BM2012:MRB113	JX145751*	JX145900*	JX145677*
*Myrmarachne* (s. lat.) *helensmithae* Pekár, 2017	P+2017:Myrm13		KT364838*	KT364800*
*Myrmarachne* (s. lat.) *macaulayi* Pekár, 2017	P+2017:Myrm17		KT364842*	KT364803*
*Myrmarachne* (s. lat.) *milledgei* Pekár, 2017	P+2017:Myrm18		KT364843*	
*Myrmarachne* (s. lat.) *zabkai* Pekár, 2017	P+2017:Myrm20		KT364844*	KT364804*

## Phylogeny

**Figures 1–4.**
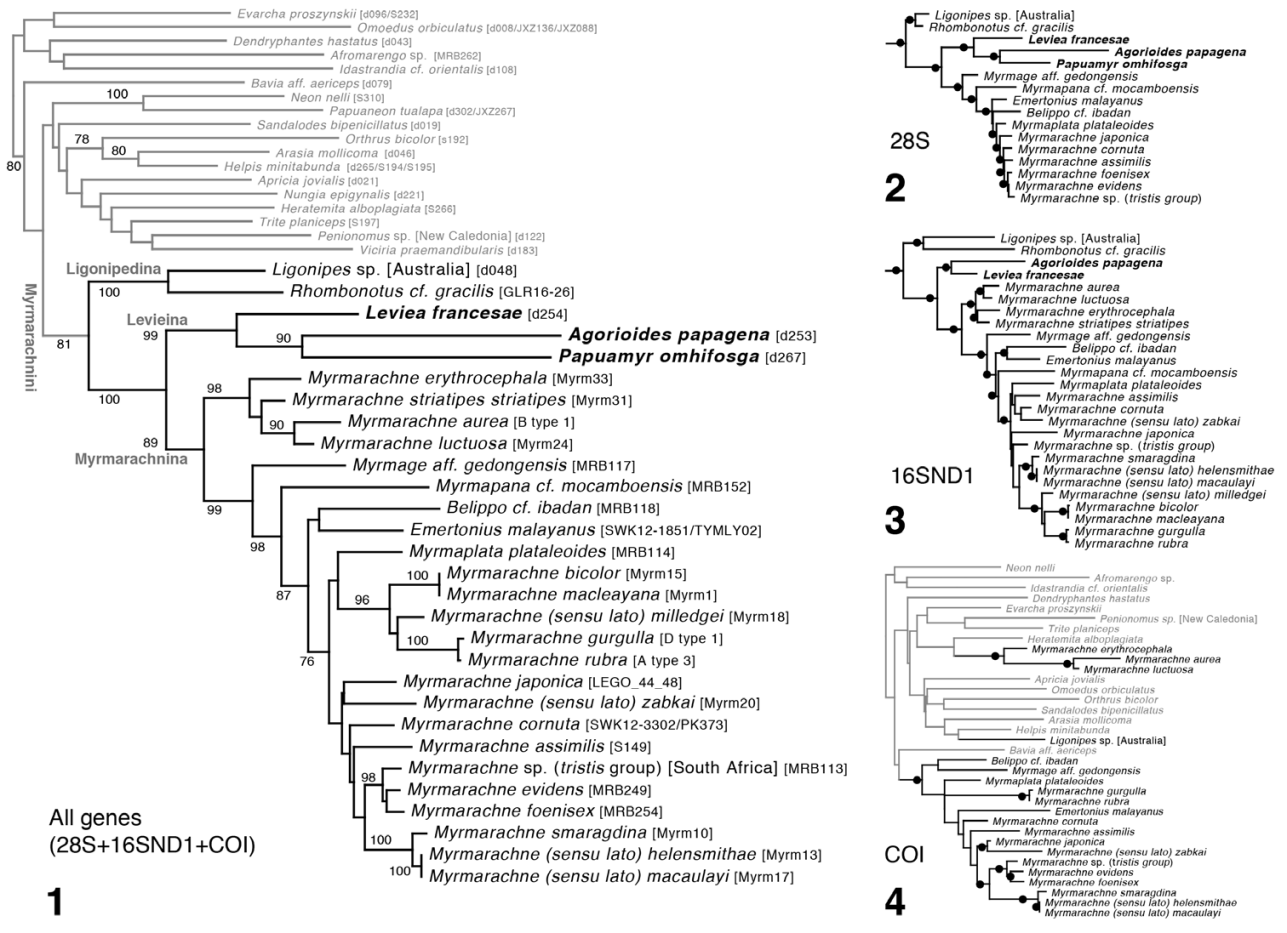
Maximum likelihood phylogenetic trees from IQ–TREE analyses. Appended to taxon names are the identification codes of voucher specimens used (see Table 1) **1** phylogeny from all 4 genes concatenated; bootstrap percentages shown if ≥ 75% **2** phylogeny from 28S analyzed alone **3** 16SND1 alone **4** COI alone.

The phylogenetic tree (Fig. 1) shows three major groups of myrmarachnines: *Ligonipes* plus *Rhombonotus*, the *Leviea* group of genera, and *Myrmarachne* sensu lato. We consider these to represent three subtribes, as described below. The three subtribes and several subgroups of the Myrmarachnina are well supported according to bootstrap values (Fig. 1) and independent support by both 28S (Fig. 2) and 16SND1 (Fig. 3). As expected from previous studies (Hedin and Maddison 2001; Maddison et al. 2008, 2014; Bodner and Maddison 2012), the gene COI on its own (Fig. 4) has discordant results, scattering the myrmarachnines and with only 53% of myrmarachine nodes in agreement with the all genes tree (compared to 87% and 69% for 28S and 16SND1, respectively). Within the Myrmarachnina, a clade of Australian *Myrmarachne* species (the *erythrocephala* group) stands as sister group to the remainder. This deep split between a handful of Australian species and the rest of the Myrmarachnina is reflected as well in the results of Ceccarelli and Crozier (2007) and Pekár et al. (2017). These results suggest that the Myrmarachnini, and the Myrmarachnina, radiated initially in Australasia, as expected given that most astioid diversification is Australasian (Bodner and Maddison 2012). Our results differ from the morphological results of Edwards and Benjamin (2009) in placing *Myrmapana* outside the clade containing the bulk of Old World species, and in placing *Belippo* within *Myrmarachne* itself (as it had been conceived in 2009).

Morphological synapomorphies are not known for the Levieina, except possibly the ectal spur on the paturon of the chelicera, and yet this group is well supported by the molecular data. Its diversity of body forms arguably exceeds that of the much more species-rich Myrmarachnina, and its molecular divergences are as deep. These suggest the levieines are an old radiation, with possibly many more species to be discovered in Australasia. Among forms we have seen (but do not describe here) are two more species of *Leviea* and three other species that may belong to *Papuamyr*, one of which is beetle-like.

The sister group relationship between *Agorioides* and *Papuamyr* is well supported in the molecular phylogeny, but we know of no morphological traits that support it. Indeed, some notable morphological characters are variable within the Levieina and discordant with the phylogeny (Ligonipedina, ((*Leviea*, (*Agorioides*, *Papuamyr*)), Myrmarachnina)). These include the antlike body (absent in *Leviea* but present in Ligonipedina, *Agorioides*, *Papuamyr*, Myrmarachnina, and absent outside the Myrmarachnini), the ectal spur on the paturon (present in *Leviea*, *Papuamyr*, and some Myrmarachnina), the RTA having a ventral flange (present in *Ligonipes*, *Leviea*, *Papuamyr*, and various Myrmarachnina, e.g., Wanless 1978 figure 3F), and the swollen first legs (absent in *Leviea*, femur-only in *Agorioides*, present in *Papuamyr* and the Ligonipedina). The lack of an antlike body in *Leviea* is most parsimoniously interpreted as a loss, given the antlike bodies in almost all other myrmarachnines, including its sister group, its first cousin (Myrmarachnina), and its second cousin (Ligonipedina). A loss in *Leviea* is perhaps not surprising, given that they are known from high elevation (2320–3700 m). In these cool mossy forests, ants are less visible and less diverse than at lower elevations (Orivel et al. 2018).

## Taxonomy

### Tribe Myrmarachnini Simon, 1901

Toxeae, Toxeinae FO Pickard-Cambridge, 1900 (replaced due to synonymy of the type genus; see Maddison 2015).

Myrmarachneae Simon, 1901

The Myrmarachnini are characterized by antlike bodies and distinctive genitalia (Edwards and Benjamin 2009). The palp has a round bulb and an immovable embolus whose terminal loop comes loose from the periphery and passes in front of (i.e., ventral to) the tegulum before terminating. The RTA is twisted and bent, frequently with a ventral flange that is sometimes developed into a distinct apophysis. The characteristic female genitalia (Wanless 1978 figure 3E) have separate atria that lie posteriorly, and lead to membranous copulatory ducts (Edwards and Benjamin 2009, character 8 state 1), sometimes irregular and voluminous, which eventually make their way to the posterior margin. The duct then narrows into a sclerotized spermathecal complex (Edwards and Benjamin 2009) that first takes the form of a narrow tube as it proceeds forward along the midline and before expanding to a bulb when it is are anterior to the atria. Prószyński (2016) considers the sclerotized tube to be part of the spermatheca, which are thus “pipe-like” in shape, while Edwards and Benjamin (2009) consider the tube to be part of the copulatory duct.

We have chosen to divide the tribe into three subtribes to reflect the group’s diversity, and to provide a formal name for the clade that would otherwise have only the informal name “*Myrmarachne* sensu lato”. A formal name for the clade is particularly urgent, now that the generic name “*Myrmarachne*” has lost its biological meaning following Prószyński’s (2016) separation of twelve genera from *Myrmarachne*. Our results indicate that *Myrmarachne* in Prószyński’s (2016) sense is not a monophyletic group. This is not surprising, given that Prószyński’s dismantling of the genus was done without considering synapomorphies, quantitative analysis, or previously published phylogenetic analyses (Ceccarelli and Crozier 2007; Edwards and Benjamin 2009; Merckx et al. 2015), but rather by carving out some groups based on few characters. At present, the word “*Myrmarachne*” means merely “The set of Myrmarachnina species that remain in *Myrmarachne*, either because a more specific place for them has not been chosen, or because they are related to the type species *Myrmarachnemelanocephala* MacLeay, 1839”. (We do not have molecular data from *M.melanocephala*, and Prószyński’s account does not guide us as to where in Fig. 1 it might fall.) Because of uncertainty about Prószyński’s new arrangement, Yamasaki et al. (2018) chose to ignore it and describe new species under the old (broader) concept of the genus *Myrmarachne*. We are now in the unsatisfactory situation in which a generic name has two meanings: in our current taxonomy (e.g., as documented in the World Spider Catalog, 2018), it refers to an unnatural conglomerate, but informally (when we use the phrase *Myrmarachne* sensu lato), it refers to a natural group (when *Bocus* and *Belippo* are included). The informal use should be avoided, as it misleads us into thinking we are relieved of the responsibility of repairing *Myrmarachne*.

While we could synonymize all Myrmarachnina back into a single genus, we have chosen not to change the current generic arrangement pending further study. The subtribe is diverse enough that it will almost certainly be split into multiple genera eventually, though possibly very differently than in Prószyński’s arrangement. (The *erythrocephala* group, for instance, could be its own genus, unusual among Myrmarachnina in retaining the excavate male chelicerae possibly ancestral for the tribe; see Pekár et al.’s (2017) figures 5H, 7E, and 13F.) By describing the subtribe, we provide a name for the clade that is a stable alternative to the phrase “*Myrmarachne* sensu lato”. Because of the lack of clear meaning of “*Myrmarachne*”, all of the species described by Pekár et al. (2017) and Yamasaki et al. (2018) would be better considered Myrmarachnina incertae sedis than members of *Myrmarachne*, but our nomenclature does not permit a species to be without generic placement. For this reason, we list their generic placement explicitly as “*Myrmarachne* (sensu lato)”.

### Subtribe Myrmarachnina Simon, 1901, new rank

Contained genera – *Belippo* Simon, 1910; *Bocus* Peckham & Peckham, 1892; *Emertonius* Peckham & Peckham, 1892; *Hermosa* Peckham & Peckham, 1892; *Myrmage* Prószyński, 2016; *Myrmagua* Prószyński, 2016; *Myrmanu* Prószyński, 2016; *Myrmapana* Prószyński, 2016; *Myrmapeni* Prószyński, 2016; *Myrmaplata* Prószyński, 2016; *Myrmarachne* MacLeay, 1839; *Myrmatheca* Prószyński, 2016; *Myrmele* Prószyński, 2016; *Panachraesta* Simon, 1900; *Toxeus* CL Koch, 1846.

**Diagnosis and synapomorphies.** A synapomorphy of this subtribe is the form of the male chelicerae: projecting forward but at most only slightly divergent; front surface usually flat. Distinguishing them from the ligonipedines and some levieines is the thinness of the first leg of the male: the patella through tarsus approximately the same thickness as that of other legs and of female.

### Subtribe Ligonipedina Simon, 1901, new rank

Ligonipedeae Simon, 1901

Contained genera – *Damoetas* Peckham & Peckham, 1886, *Judalana* Rix, 1999, *Ligonipes* Karsch, 1878, *Rhombonotus* L Koch, 1879.

**Diagnosis and synapomorphies.**Edwards and Benjamin (2009) indicate two distinct synapomorphies for the Ligonipedina: a bend in the duct-like portion of the spermathecal complex and medially placed atrial rims. These characters provide only weak support for the group as a whole, though if *Damoetas* were removed, the remaining genera would form a tight group well supported by a distinctive feature: the male palp laterally compressed, and with a groove in tegulum in which the last loop of the embolus rests as it passes over the tegulum. *Ligonipes*, *Rhombonotus*, and *Judalana* are also united by the first leg being swollen, especially patella and tibia, with ventral fringe of hairs.

### Subtribe Levieina, new subtribe

Type genus: *Leviea* gen. n.

Contained genera – *Agorioides* gen. n., *Leviea* gen. n., *Papuamyr* gen. n.

**Diagnosis and synapomorphies.** There are no unambiguous morphological diagnostic traits known for the levieines, though there are molecular traits. We tentatively suggest as a synapomorphy of this group the ectal spur on the male cheliceral paturon (white arrow in Figs 13, 29, 71, 72, 83, 84). It is absent in *Agorioides*, in which, judging by phylogenetic position, the spur could be secondarily lost. Such a spur is seen also in some Myrmarachnina (see illustrations of Wanless 1978): *Belippo* spp., *Hermosaandrewi* (Wanless, 1978), *Myrmarachnelesserti* Lawrence, 1938, and *Myrmeleeugenei* (Wanless, 1978). Given the apparently scattered distribution of this trait, it could be convergent with that in levieines. Other than this, no morphological traits are known to unite levieines. In the gene 28S, the three levieine genera are unique among myrmarachnines in having a G at our alignment’s positions 579, 646, 746, 754, and a T at 814; these correspond to sites 548, 614, 698, 706, and 761 in the unaligned *Levieafrancesae* 28S sequence.

#### Leviea gen. n.

Taxon classificationAnimaliaAraneaeSalticidae

http://zoobank.org/DF5C50F5-4A8C-4DA0-A1A0-E0594BE01745

##### Type species.

*Levieaherberti* sp. n.

##### Etymology.

This distinctive genus is named in honour of Herbert Walter Levi, his partner Lorna Rose Levi, and their daughter Frances Levi. Dr. Levi (or, Herb, as he humbly preferred to be known by) was one of the grand arachnologists of the twentieth century, describing over 1200 species of spiders, mentoring many subsequent leaders of the field, and curating one of arachnology’s most important museum collections (Leibensperger 2016). Lorna collaborated in his work in many ways, co-authoring the classic book *Spiders and their kin* (Levi and Levi 1968), which introduced the first author of this paper to spider diversity. Frances accompanied them in the field and carried on an interest in woven creations. Their contributions, both personal and scientific, will long be remembered (Maddison 2014a, b; Leibensperger 2016). The Levis pronounced the vowels of their name approximately as their IPA equivalents (*e* as in *Ed*, *i* as in *eat*). The last three letters of *Leviea* are to be pronounced as three separate vowels (as their IPA equivalents, i-e-a). The name is to be treated grammatically as feminine.

##### Diagnosis.

The form of the body is not in the least bit reminiscent of an ant, beetle or wasp, unlike other myrmarachnines. Instead, the body is of standard salticid form (e.g., *Icius* Simon, 1876, *Salticus* Latreille, 1804), somewhat glabrous, with chevron markings. Two features possibly retained from antlike ancestors are a female palp that is widened and somewhat dorso-ventrally flattened, and the many long macrosetae on the first tibia in two of the *Leviea* species. The male embolus is distinctive for ending broadly, not tapering to a point. As in *Papuamyr*, there is an ectal spur on the paturon (white arrow in Figs 13, 29).

#### Leviea herberti sp. n.

Taxon classificationAnimaliaAraneaeSalticidae

http://zoobank.org/3FC00E58-07A2-4551-A6EF-C6461EF46A55

Figs 5–20 , 90

##### Type material.

*Holotype*: male, specimen PNG2008-0360 in UBC–SEM, with data PAPUA NEW GUINEA: Enga Province: Kai-ingri. 5.579 S 143.053 E. 3240 m a.s.l. 7–9 July 2008. W Maddison & Manisé Kulé leg. WPM#08-005. Beating understory of *Phyllocladus* forest. *Paratype*: female, specimen PNG2008-0370, with same data as holotype, in UBC–SEM.

##### Etymology.

Named in honour of Dr Herbert W Levi.

##### Diagnosis.

**Figures 5–20.**
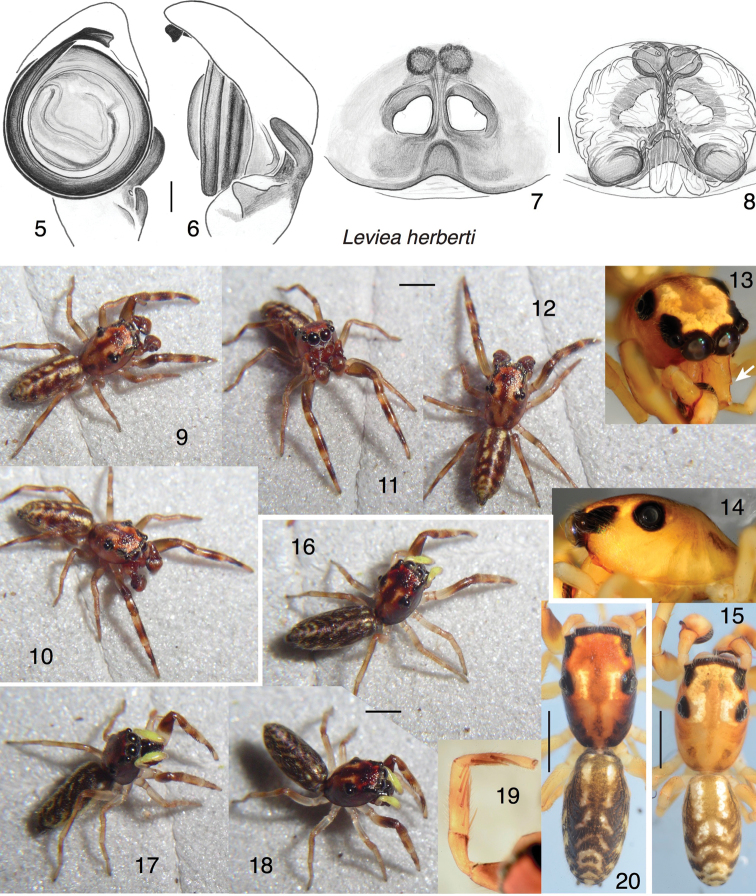
*Levieaherberti* sp. n., holotype male and paratype female. **5, 6** Left palp **5** ventral view **6** retrolateral view **7** epigyne, ventral view (paratype female) **8** cleared vulva, dorsal view (same female) **9–15** holotype male **13** face; arrow shows ectal spur on paturon **14** side of carapace **15** habitus dorsal view; two photographs joined **16–20** paratype female **19** prolateral view of first leg **20** habitus dorsal view; two photographs joined. Scale bars: 0.1 mm (on genitalia); 1.0 mm (on bodies).

**Figures 21–33.**
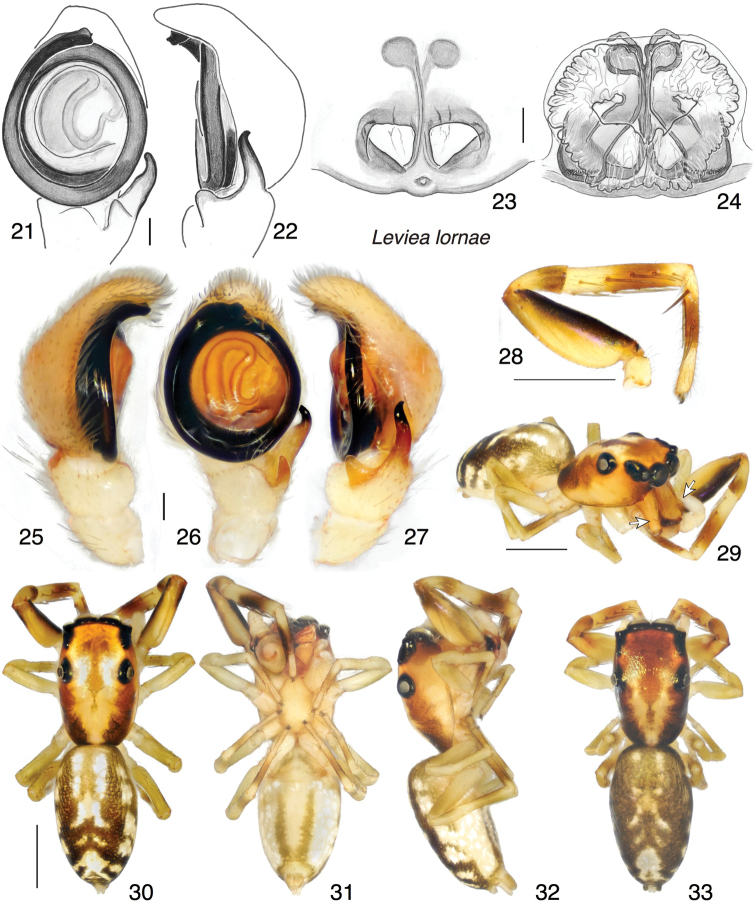
*Leviealornae* sp. n., holotype (**25–32**) and paratypes. **21, 22** Left palp **21** ventral view **22** retrolateral view **23** female epigyne, ventral view **24** cleared vulva, dorsal view **25** left palp, prolateral view **26** same, ventral view **27** same, retrolateral view **28** first leg, prolateral view **29** male habitus, oblique lateral-frontal view; arrows shows ectal spurs on paturon **30** dorsal view **31** ventral view **32** lateral view **33** female habitus dorsal view. Scale bars: 0.1 mm (on genitalia); 1.0 mm (otherwise).

Body somewhat smaller, less elongate, than the other species of *Leviea*, and with fewer (3 or 3.5 pairs) ventral macrosetae on first tibia. The robust dorsal branch of the RTA ends bluntly like a thumb, unlike the tapering and dorsally pointing tip of the other species. Bulb ca. 90° further rotated than in *L.lornae* and *L.francesae*, as indicated by the spermophores (Figs 5, 21, 38).

##### Description.

*Male* (holotype). Carapace length 1.82; abdomen length 1.96. Carapace (Figs 12, 14, 15): Of typical salticid shape, without constrictions or thoracic hump. Chelicera (Fig. 13): Vertical, simple except for a small ectal spur on the paturon. At least five retromarginal teeth. Palp (Figs 5, 6). With round bulb. Embolus circling 1.8 times around, ending in broad tip. RTA appearing as a finger and thumb (dorsal apophysis and ventral flange, respectively), with the dorsal apophysis pointing somewhat ventrally. Legs unremarkable, first somewhat more robust. First tibia with three pairs of ventral macrosetae, of normal length (right tibia bears an extra small distal anterior macroseta). Colour in life (Figs 9–12): Markings are muted beige to reddish brown, with the digestive diverticula in the ocular area providing two pale stripes that continue as pale areas on the thorax and chevrons on the abdomen. Colour in alcohol (Fig. 13–15): Body and appendages honey coloured, with indistinct dark annuli on the legs, and chevrons on the abdomen. Underside of abdomen with two longitudinal dark stripes.

*Female* (paratype, specimen PNG2008-0370). Carapace length 1.89; abdomen length 2.16. Carapace, legs, abdomen substantially as in male (Figs 16–18, 20), except for the less robust first legs. First leg tibia with seven ventral macrosetae (three pairs plus one prolateral-distal) (Fig. 19). Epigyne (Figs 7, 8): Of fairly typical myrmarachnine form. RTA pocket more distinct and larger than in *L.lornae*. Colour (Figs 16–18, 20): As in male, but with body and chelicerae darker, and palps bright yellow in life.

#### Leviea lornae sp. n.

Taxon classificationAnimaliaAraneaeSalticidae

http://zoobank.org/2D163627-1C71-42C8-B503-4284E877F4DB

Figs 21–33 , 90

##### Type material.

*Holotype*: male specimen in RBINS, with data PAPUA NEW GUINEA: Chimbu Province, Mount Wilhelm, Pinde-Yaunde Lake 5.78S, 145.06E. 3700 m a.s.l. 4 October 2013. Sub-alpine forest near the limit tree vegetation. Gewa, Damag, Novotny, Leponce leg. #P3633 Beating understory. *Paratypes*: 5 males (from collection events #P3609, #P3631, #P3634, #P3637, #P3640) 4 females (from collection events #P3598, #P3632, #3619, #P3633,) in RBINS with same data as the holotype. 3 males and 3 females (events #P3619, #P3637) in UBC–SEM with same data as the holotype. 2 males (events #P3232, #P3241), 1 female (event #P3237) in RBINS with data PAPUA NEW GUINEA: Madang Province: Kombunomambuno 5.80S, 145.07E. 3200 m a.s.l. 4 October 2013. Upper montane forest. Dahl, Kaupa, Novotny, Leponce leg. Beating understory. 4 males 4 females in HNHM with data PAPUA NEW GUINEA: Madang Province: Mt Wilhelm, Moss forest at the meteorological station of Kambugomanbuno [sic!]. 3018 m a.s.l. 1968.09.17. János Balogh leg. NGM C26 Beating. (HNHMAraneae-9251, 9252, 9253, 9254).

##### Etymology.

Named in honour of Lorna Levi.

##### Diagnosis.

Larger and more slender than *L.herberti*, with more (5 pairs) ventral macrosetae on first tibia. In these features it resembles *L.francesae*, from which it differs in details of the palp: dorsal branch of RTA slender and distal-pointing; bulb rounder than *L.francesae*, less rotated than *L.herberti*. Epigyne with openings further posterior than those of *L.herberti*, and smaller RTA pocket at rear margin.

##### Description.

*Male* (paratype in UBC–SEM). Carapace length 2.13; abdomen length 2.35. Carapace (Figs 30, 32): Of typical salticid shape, without constrictions or thoracic hump. Narrower than *L.herberti*. Chelicera (Fig. 29): Vertical, with ectal spur on the paturon. At least five retromarginal teeth. Palp (Figs 21, 22, 25–27): Bulb round. Embolus circles 1.7 times around, ending in broad tip. RTA with dorsal branch slender, pointed, pointing distally. First leg tibia with five pairs of ventral spines (Fig. 28). Colour in alcohol (Figs 29–32): Markings much like those of *L.herberti*, though body darker. Honey coloured with black around eyes, dark brown on sides of thorax, and extensive black on abdomen. First leg with dark patches, appearing annulate.

*Female* (paratype in UBC–SEM). Carapace length 1.89; abdomen length 2.45. Carapace (Fig. 33): As in male. First leg tibia with five pairs of ventral spines. Epigyne (Figs 23, 24): With triangular openings pointing to the posterior. Colour in alcohol (Fig. 33): As in male, but with weaker dark patches on first legs.

##### Additional material.

**Figures 34–37.**
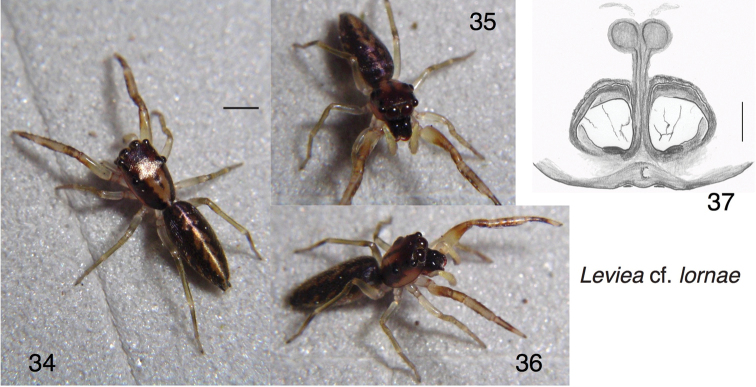
Levieacf.lornae, female from Mt. Gahavisuka. **34–36** Living specimen **37** epigyne, ventral view. Scale bars: 0.1 mm (on epigyne); 1.0 mm (otherwise).

The range of this species may extend to the east. A female (specimen PNG2008-3321 in UBC–SEM) slightly differing in epigynal structure is tentatively assigned to this species. It is pictured in Figs 34–37, and has data: PAPUA NEW GUINEA: Eastern Highlands Province: Mt Gahavisuka Provincial Park. S 6.015 E 145.412. elev. 2320 m a.s.l. 1–2 August 2008. W Maddison leg. WPM#08-025.

#### Leviea francesae sp. n.

Taxon classificationAnimaliaAraneaeSalticidae

http://zoobank.org/CED5C66E-1FD2-4012-A3C5-736477DDAA0B

Figs 38–41 , 90

##### Type material.

*Holotype*: male, DNA voucher d254, in UBC–SEM, with data PAPUA NEW GUINEA: Enga Province: Kai-ingri. 5.574 S 143.048 E. 3315 m a.s.l. 5–8 July 2008. W Maddison leg. WPM#08-004. *Paratype*: one male, in UBC–SEM, with data Papua New Guinea: Enga Province: Kumul Lodge @ foot of Mt Hagen, 05.47.548 S 143.58.761 E. 2700 m. 5.xii.2006. Balke & Kimbel leg. (PNG124).

##### Etymology.

Named in honour of Frances Levi.

##### Diagnosis.

Larger and more slender than *L.herberti*, with more (5 pairs) ventral macrosetae on the first tibia. In these features it resembles *L.lornae*. Differs from both *L.herberti* and *L.lornae* in details of the palp: dorsal branch of RTA swollen basally; bulb oval rather than circular, less rotated than *L.herberti*.

##### Description.

**Figures 38–41.**
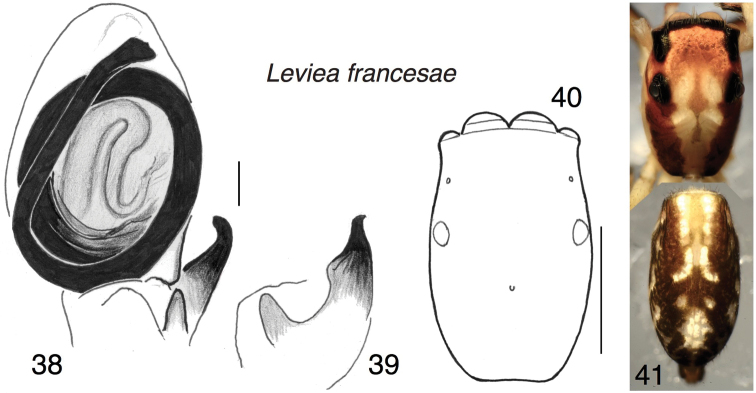
*Levieafrancesae* sp. n., holotype male. **38, 39** Left palp **38** ventral view **39** retrolateral view of tibia **40** carapace **41** habitus dorsal view; two photographs joined. Scale bars: 0.1 mm (on palp); 1.0 mm (on carapace).

*Male* (holotype). Carapace length 2.08; abdomen length 2.38. Carapace (Fig. 41): Like that of *L.lornae*. Chelicera: Ectal spur on paturon small. Five retromarginal teeth. Palp (Figs 38, 39): Bulb compressed slightly laterally to be oval rather than circular. Embolus thick, circling 1.7 times around. First leg tibia with five pairs of ventral spines. Colour in alcohol (Fig. 41): much like that of *L.herberti*.

*Female*. Unknown.

#### Agorioides gen. n.

Taxon classificationAnimaliaAraneaeSalticidae

http://zoobank.org/2CCDC6C4-BBB2-42E1-92F0-8B97BD59EFBC

##### Type species.

*Agorioidescherubino* sp. n.

##### Etymology.

Named for the spiders’ superficial resemblance to *Agorius* Thorell, 1877.

##### Diagnosis.

Antlike, with concave-sided carapace, swollen first femur, a long ocular quadrangle, long fourth trochanters, and a spinose first tibia. The carapace is sunken inward (concave) between the PME and PLE, leaving the PLE on prominent tubercles, and yielding a constriction that resembles that of hisponine salticids. The femur of the first leg is shaped like a bird’s lower leg (“drumstick”), swollen in the proximal half but thin distally. The length of the ocular quadrangle is distinctly more than half the length of the carapace. The fourth trochanter is unusually long, longer than either the coxa or the fourth tarsus. Unlike *Leviea* and *Papuamyr*, the paturon of the chelicera lacks an ectal spur, and the first tibia has many pairs of long macrosetae; both of these features can be found in some species of Myrmarachnina.

##### Remarks.

The two species described are closely similar, but distinct in the form of the palp, shape of the carapace, and in colour. They were found only seven km apart, but at distinct elevations (570 m vs. ca. 1000 m). In other salticid genera, closely related species have been observed to segregate along such an elevational gradient in the same area (e.g., *Cucudetazabkai* Maddison, 2009 vs. *Cucudetauzet* Maddison, 2009 at 1170 m vs. 1450 m [Maddison 2009]).

#### Agorioides cherubino sp. n.

Taxon classificationAnimaliaAraneaeSalticidae

http://zoobank.org/3AFED112-EFEE-4DE7-91C8-C3C1246D9CC4

Figs 42–50 , 90

##### Type material.

*Holotype*: male in UBC–SEM, specimen code PNG2008-2854, with data PAPUA NEW GUINEA: Southern Highlands Province: Putuwé, junction of Lagaip & Uruwabwa Rivers. 5.231 S 142.532 E. 570 m a.s.l. 23–26 July 2008. W Maddison & Luc Fimo Tuki leg. WPM#08-019. On leaf litter.

##### Etymology.

In the Levis’ country home they hosted many animals, domesticated and not. One of these was a dog of multifarious ancestry, Cherubino, named after the character in Mozart’s opera *The Marriage of Figaro*. The spider resembles the dog in having a hairy, grizzled appearance.

##### Diagnosis.

**Figures 42–50.**
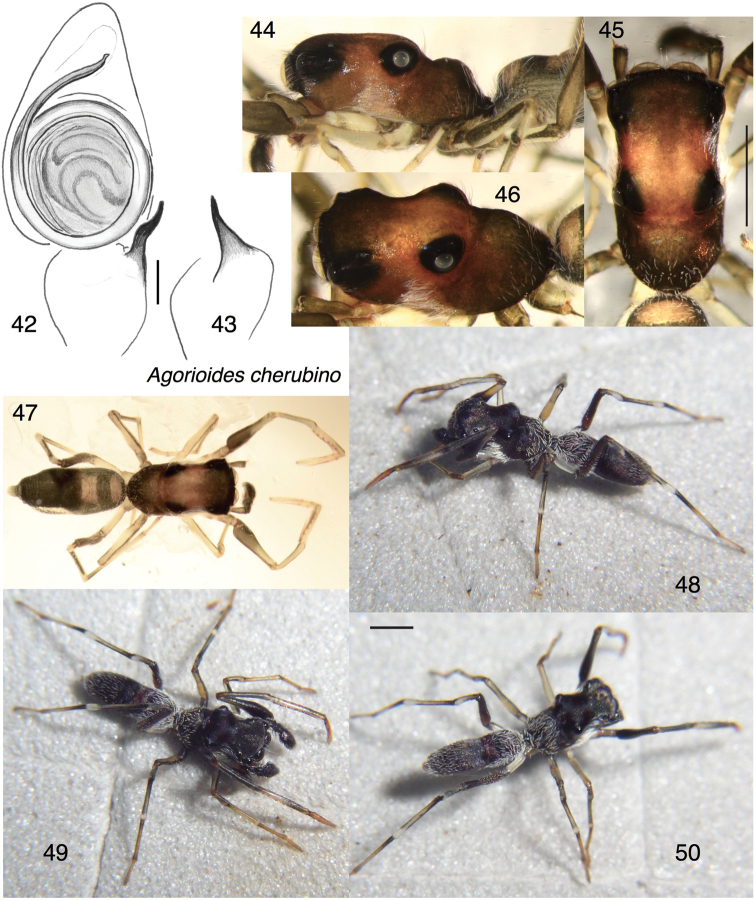
*Agorioidescherubino* sp. n., holotype male. **42, 43** Left palp **42** ventral view **43** retrolateral view of tibia **44–46** carapace **44** lateral view **45** dorsal view **46** oblique dorsal-lateral view **47** male habitus, dorsal view **48–50** living specimen. Scale bars: 0.1 mm (on genitalia); 1.0 mm (otherwise).

Differs from *A.papagena* in having the tibia of the male palp distinctly narrower than the cymbium (Figs 42 vs. 51), the cephalic area distinctly higher (Figs 44 vs. 53), and in the black body with a dusting of white setae. The bulb of the palp is rotated slightly more than in *A.papagena* (see Diagnosis for that species).

##### Description.

*Male* (holotype). Carapace length 2.16; abdomen length 2.27. Carapace (Figs 44–50): Strangely shaped, as described for the genus, with a constriction between the PME and PLE. Thoracic hump prominent (Fig. 44). Ocular quadrangle occupies more than half of the length of the carapace. Clypeus extremely narrow. Chelicera: Vertical, simple. Teeth not examined for fear of damaging the specimen. Palp (Figs 42, 43): Embolus wrapping around bulb more than once; RTA simple and unbranched. Legs with relatively few short setae, except a greater density yields weak fringes beneath metatarsi and tarsi 2–4 and tibiae 3–4. First tibia with seven pairs of long ventral macrosetae; first metatarsus with two pairs. First femur distinctly swollen in proximal half, shaped like a bird leg’s drumstick (Figs 47, 49). Fourth trochanters distinctly longest, longer than the fourth tarsi (Figs 44, 48). Colour in life (Figs 48–50): Black body with fully or partly erect white setae on the sides and back of the carapace, the abdomen, and the femora of the fourth legs. First and fourth femora black; other segments translucent white to honey with darker lines or patches. Colour in alcohol (Figs 44–47): Carapace brown with black around eyes. Abdomen black except paler around the constriction and anteriorly. Clypeus dark, with only a few setae. Chelicerae honey coloured. Palp black. Legs pale except black on proximal half of first femur, brown to black fourth femur, and black lines on anterior faces of legs 2–4 and posterior faces of legs 3 and 4.

*Female.* Unknown.

##### Additional material examined.

One penultimate instar male (specimen PNG2008-2765, in UBC–SEM), with same data as holotype, also from leaf litter. It has a black body and leg markings as in holotype, but is more glabrous, with only a few white setae.

#### Agorioides papagena sp. n.

Taxon classificationAnimaliaAraneaeSalticidae

http://zoobank.org/15AE375D-818D-42F9-AE96-3A5B9097F659

Figs 51–59 , 90

##### Type material.

*Holotype*: male in UBC–SEM, specimen code PNG2008-1706 and DNA voucher code d253, with data PAPUA NEW GUINEA: Southern Highlands Province: Tualapa, near Wanakipa. 5.283 S 142.498 E. 1000–1100 m a.s.l. 11–22 July 2008. W Maddison & Luc Fimo Tuki leg. WPM#08-008. Forest interior and riverside on leaf litter.

##### Etymology.

The Levis’ love of opera was reflected in their animals’ names, including their dog Papagena, named after the character in Mozart’s *The Magic Flute*. The spider does not look like the dog or the opera character. Although the holotype is a male, the species is named for the female that remains to be found.

##### Diagnosis.

**Figures 51–59.**
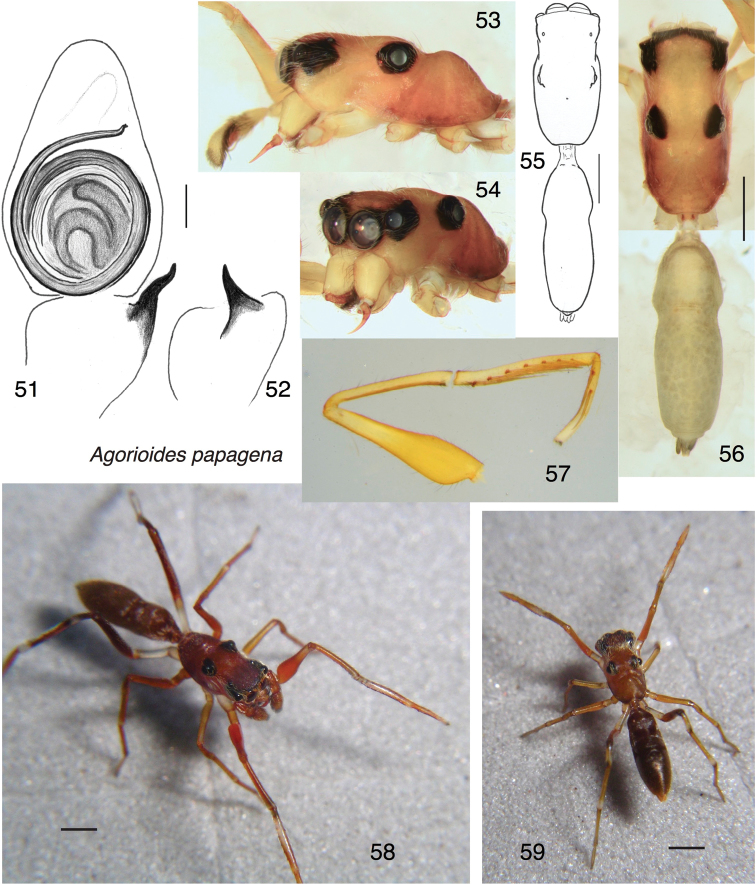
*Agorioidespapagena* sp. n., holotype male except for **59** (juvenile). **51, 52** Left palp: **51** ventral view **52** retrolateral view of tibia **53** Carapace, lateral view. **54** Frontal-lateral view **55, 56** dorsal habitus **57** first leg, prolateral view (two photographs joined at broken patella-tibia joint) **58** living holotype **59** juvenile from type locality. Scale bars: 0.1 mm (on genitalia); 1.0 mm (otherwise).

Differs from *A.cherubino* in having the tibia of the male palp almost as wide as the cymbium (Fig. 51), flatter profile of the carapace (Fig. 53), and a distinctly orange body and legs. The bulb of the palp is rotated less in *A.papagena* than in *A.cherubino*, as seen by the orientation of the spermophores in Fig. 51 versus Fig. 42. Although this could be due to a slight expansion of the palp of the one known specimen of *A.papagena*, suggested by the offset of the tip of its embolus from the cymbial apical groove, this offset is of lesser angle than the difference in spermophore orientation.

##### Description.

*Male* (holotype). Carapace length 2.84; abdomen length 3.24. Structure of body, legs as in *A.cherubino* (Figs 53–57), with first leg having swollen femur (Figs 57, 58). Chelicera (Fig. 54): Vertical, though robust. Four retromarginal teeth. Palp (Figs 51, 52): Embolus wrapping around bulb more than once; RTA simple and unbranched. Tibia distinctly wider than in *A.cherubino*. Colour in life (Fig. 58): Orange, darkening to brown on the abdomen and with black around the eyes. Swollen femur of the first leg particularly bright orange. Some partially-erect white setae on carapace sides beneath the eyes, on thorax, and on fourth femora, but not as dense or distinct as in *A.cherubino*. Colour in alcohol (Figs 53, 54, 56, 57): Except for black around the eyes, carapace, legs and palpi are orange, darkest on the thorax and palest at the leg tarsi. Abdomen a muted orangish gray.

*Female.* Unknown.

##### Additional material examined.

One juvenile (specimen PNG2008-1676, in UBC–SEM, Fig. 59), similarly coloured, with same data as holotype, on leaf litter.

#### Agorioides

Taxon classificationAnimaliaAraneaeSalticidae

sp.

Figs 60–65 , 90

##### Description.

A single large female from the Muller Range is clearly an *Agorioides* by carapace shape and long fourth trochanters (the first legs are missing), but is not formally described here because the specimen is missing most of its legs. It seems likely to represent a distinct species, as it has a carapace that is flatter (Fig. 65) than that of the two described above. It is notably larger than *A.cherubino* (carapace 3.00, abdomen 3.43). Its epigyne is typical myrmarachnine, with the RTA pocket displaced anteriorly (Figs 62, 63). Its data are: PAPUA NEW GUINEA: Western Province: Muller Range, Camp 1, Gugusu. 05.7292S, 142.2633E. 515m elev. 4–11 September 2009. I Agnarsson leg.

**Figures 60–65.**
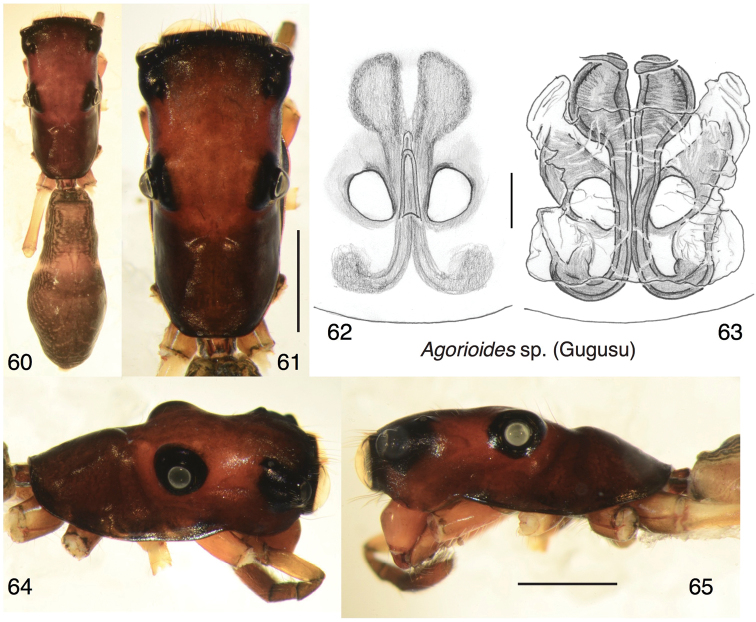
*Agorioides* sp., female from Gugusu, Muller Range. **60** Habitus, dorsal view **61** carapace, dorsal view **62** epigyne, ventral view **63** cleared vulva, dorsal view **64** carapace, oblique dorsal-lateral view **65** carapace, lateral view. Scale bars: 0.1 mm (on genitalia); 1.0 mm (otherwise).

#### Papuamyr gen. n.

Taxon classificationAnimaliaAraneaeSalticidae

http://zoobank.org/D0A7CD62-1349-4A0D-858D-8EB52020A454

##### Type species.

*Papuamyromhifosga* sp. n.

##### Etymology.

A blend of Papua and the first syllable of the name of the related genus *Myrmarachne*. To be treated as feminine, as is *Myrmarachne*.

##### Diagnosis.

Small antlike salticids with somewhat swollen male first legs, vertical and excavated male chelicerae with an ectal spur on the paturon, a round bulb on the palp, and a relatively long RTA. In contrast, the *Ligonipes* group of genera has the bulb of the male palp compressed laterally (Davies and Żabka 1989: figs 7 and 9), while most *Myrmarachne* males have projecting chelicerae and thin first legs. An apparent synapomorphy uniting the two *Papuamyr* species described below is a complex fold in the embolus at the point where it crosses over previous loops before terminating (see black arrows in Figs 70 and 78).

##### Remarks.

*Papuamyr* lacks the morphological diagnostic characters of the Ligonipedina and Myrmarachnina, but we have not found morphological characters that place it more clearly. The molecular data, however, are clear that *Papuamyr* is within the Levieina and a close relative of *Agorioides* (Fig. 1).

#### Papuamyr omhifosga sp. n.

Taxon classificationAnimaliaAraneaeSalticidae

http://zoobank.org/2A55E9AA-C102-4368-9D71-12E10EFCD128

Figs 66–77 , 90

##### Type material.

*Holotype*: male, specimen UBC–SEM AR00215 in UBC–SEM, with data PAPUA NEW GUINEA: Southern Highlands Province: Putuwé, junction of Lagaip & Uruwabwa Rivers. 5.231 S 142.532 E. 570 m a.s.l. 23–26 July 2008. W Maddison & Luc Fimo Tuki leg. WPM#08-019. Beating. *Paratypes*: 5 males, 5 females, 2 juveniles, with same data.

##### Etymology.

A combination of letters derived from the first letters of words in a statement of Herbert Levi’s from approximately 1985. He had forgotten to do some bureaucratic task, and in dismay, he exclaimed “Och, my head is full of spider genitalia!” Truly, it was, and for that knowledge which he conveyed to us, arachnology is forever enriched. The name is particularly apt for this species: its genitalia are distinctive and elegant, the palp having a long transparent RTA and sharply bent embolus, the epigyne an RTA pocket displaced far to the anterior.

##### Diagnosis.

Distinct for its unusual genitalia and its orange and black bicoloured body. The embolus tip extends onto the retrolateral side of the cymbium before looping back to terminate ventrally, much as seen in many amycoid salticids such as *Tartamura* (Bustamante and Ruiz 2017). Most notable, however, is the long RTA whose posterior branch extends almost to the tip of the cymbium (Fig. 67). Accordingly, the RTA pocket of the epigyne is displaced far anteriorly (Fig. 68).

##### Description.

**Figures 66–77.**
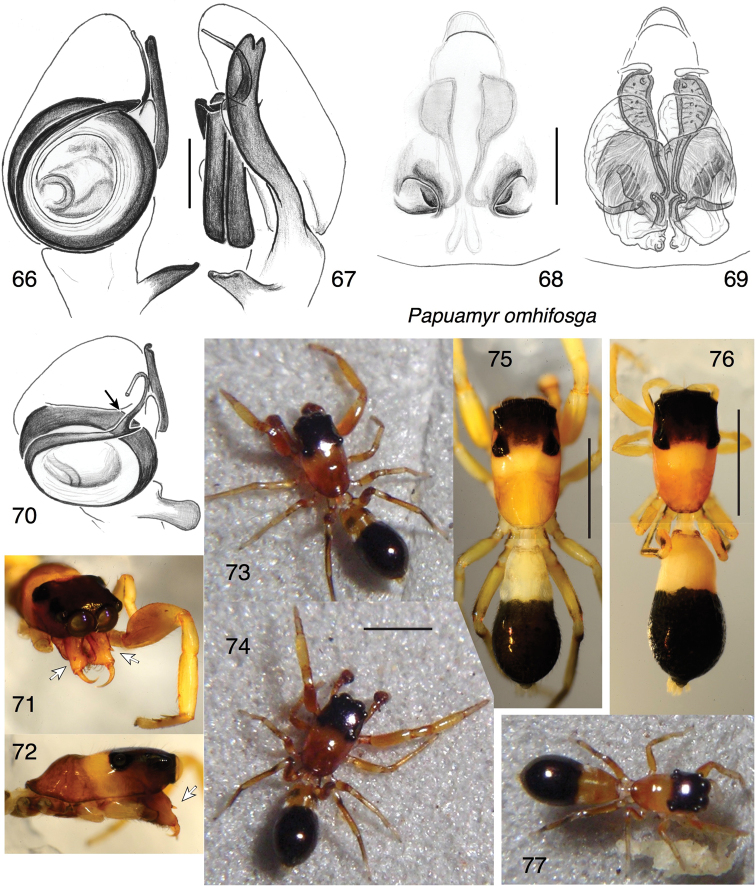
*Papuamyromhifosga* sp. n., holotype (**66, 67, 70, 75**) and paratypes. **66, 67** Left palp of holotype **66** ventral view **67** retrolateral view **68** epigyne of specimen PNG2008-2603, ventral view **69** cleared vulva of same specimen, dorsal view **70** left palp of holotype, oblique ventral-terminal view; arrow shoes bend in embolus **71** face of male, oblique frontal-lateral-dorsal view; arrows show ectal spurs on paturons **72** carapace, lateral view **73, 74** living male **75** habitus, dorsal view, holotype male **76** habitus, dorsal view, female UBC–SEM AR00216 **77** living female. Scale bars: 0.1 mm (on genitalia); 1.0 mm (otherwise).

*Male* (holotype). Carapace length 1.31; abdomen length 1.24. Carapace (Figs 71–75): Narrow and flat, with a thoracic hump but without a strong constriction (Fig. 72). Carapace glabrous and shiny, without evident microsculpture. Ocular quadrangle less than half the length of the carapace. Clypeus extremely narrow. Chelicera (Fig. 71): Excavated medially, and with an ectal spur on the paturon. Five very small retromarginal teeth. Palp (Figs 66, 67, 70): Bulb round, with embolus with a sharp bend near the terminus (black arrow in Fig. 70), at the point where it passes a previous loop, just before extending to the retrolateral side of the cymbium. Ventral lobe of RTA projects ventrally; dorsal lobe extremely long. Legs with relatively few, short setae. First legs distinctly more robust than others. First tibia with two short anterior ventral macrosetae in the distal third, and two extremely small posterior ventral macrosetae in the proximal two thirds. Colour (Figs 71–75): Orange (vibrant in life, pale in alcohol) except for the dark cephalic region, the black posterior half of the abdomen, and dark lines on legs 3 and 4.

*Female* (paratype, specimen # PNG2008-2603). Carapace length 1.13; abdomen length 1.46. Carapace (Figs 76, 77): As in male. Chelicera: At least four retromarginal teeth. Legs similar to male except for the smaller first leg. First tibia with ventral macrosetae longer than in male, of normal length. Four macrosetae on first tibia (as in male, from distal to basal one anterior- one pair - one posterior), though five in other specimens (two pairs distally, and one posterior basally). Epigyne (Figs 68, 69): Of typical myrmarachnine form (see description of the tribe), except that RTA pocket is far to the anterior. Colour (Figs 76, 77): As in male.

#### Papuamyr pandora sp. n.

Taxon classificationAnimaliaAraneaeSalticidae

http://zoobank.org/46402467-D46D-464D-99F5-2D1229893DF0

Figs 78–89 , 90

##### Type material.

Holotype: male, specimen in RBINS, with data PAPUA NEW GUINEA: Madang Province, Oromongu, 5.73S, 142.53E. 700 m a.s.l. 26 January 2014. Maurice Leponce leg. #P5097. Mixed lowland forest Mixed evergreen forest of foothills and mountains, beating. *Paratypes*: 3 males, 3 females, with same data (from collecting events: males: #P5088, #P5103, #P5110, females: #P5082, #P5071, #P5106).

##### Etymology.

From the Greek “all giving” or “all gifted”, referring primarily to the gifts given us by the Levis, most memorably the 1254 species that Herb described, and the wonderfully abundant Thanksgiving celebration dinners that Lorna put on for their students each year. It is also the name of the dog Lorna had when Herb and Lorna were married.

##### Diagnosis.

Distinct in having the thoracic hump higher than the ocular area in both sexes (Figs 84, 87, 89). Males can be recognized by the shape of the RTA, with dorsal branch shorter than in *P.omhifosga* and twisted, and a very short ventral flange (Fig. 80). Females can be recognized by the lack of anterior pocket, the relatively large spermathecae (Fig. 81), and the long lateral extension of the sclerotized ducts near the posterior margin (Fig. 82).

##### Description.

**Figures 78–89.**
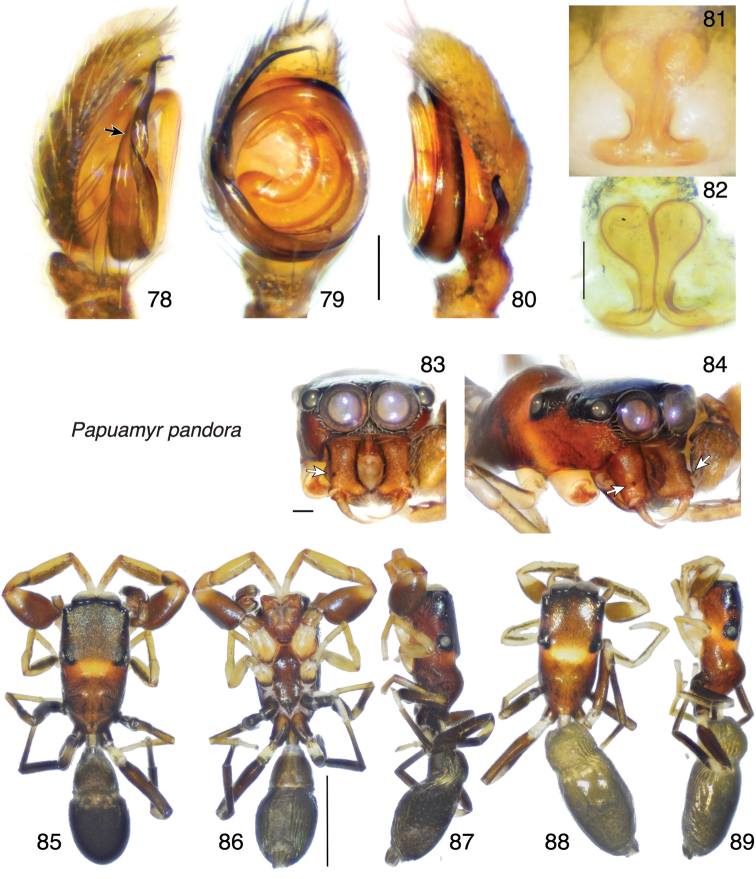
*Papuamyrpandora* sp. n., holotype, except **81, 82** and **88–89** (paratype). **78–80** Left palp **78** prolateral view **79** ventral view **80** retrolateral view **81** female epigyne, ventral view **82** cleared vulva, dorsal view **83** male carapace, frontal view **84** oblique lateral-ventral view showing patural teeth (indicated by arrows) **85** male habitus, dorsal view **86** ventral view **87** lateral view **88** female habitus dorsal view **89** lateral view. Scale bars: 0.1 mm (on genitalia and **83**); 1.0 mm (otherwise).

*Male* (holotype). Carapace length 1.52; abdomen length 1.41. Carapace (Figs 83–85): Thoracic slope with a large hump which is higher than the flat ocular area (Fig. 84). Ocular quadrangle with rugose integument (Fig. 85), approximately as long as wide, occupying half of the carapace (0.75 long). Clypeus extremely narrow (Fig. 83). Chelicera (Figs 83, 84): Excavated medially, with an ectal spur on the paturon. Palp (Figs 78–80): cymbium with 3–4 macroseta on the apical part, bulb round, embolus looped twice around it, with a twist on the prolateral side of the bulb (black arrow Fig. 78) and a slight bend at the end. Ventral flange of RTA small, a slight hump; dorsal lobe well developed, with an elongated “S” shape. Legs: Leg I robust, each segment is at least twice as wide as other legs. Other legs slender. Abdomen: pear shaped, with a slight constriction at the anterior fourth/third. Colour (Figs 85–87 in alcohol): Body dark brown with a transverse pale band in the middle of the carapace and abdomen. Coxa I, leg II, proximal half of coxa IV, patella III-IV, metatarsus III, and all tarsi pale yellow. Trochanter and femur I dark brown, patella and metatarsus I dark yellow with a dark ventral side. Leg III-IV black except the pale yellow segments mentioned earlier (Figs 85–87).

*Female*: (paratype, from collecting event #P5013). Carapace length 1.41; abdomen length 1.54. Carapace (Figs 88, 89): As in male. Chelicera: unmodified. Legs similar to male except for the less robust first leg. Epigyne (Figs 81, 82): Spermathecae large, touching each other. Sclerotized ducts of spermathecal complex oriented laterally at back margin before proceeding anterior to the fertilization duct. Colour (Figs 88, 89): As in male.

**Figure 90.**
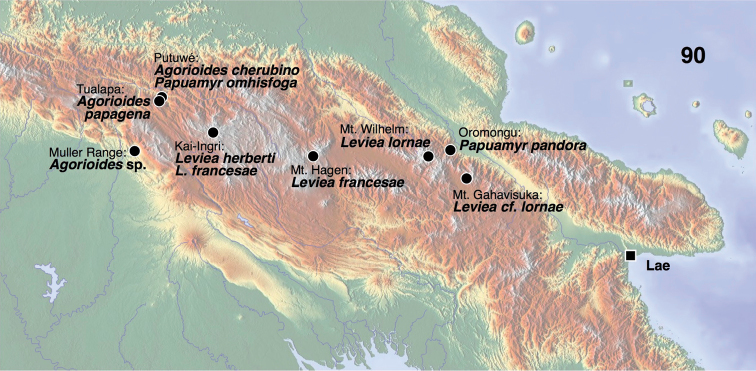
Distribution of levieines in Papua New Guinea.

## Supplementary Material

XML Treatment for Leviea

XML Treatment for Leviea herberti

XML Treatment for Leviea lornae

XML Treatment for Leviea francesae

XML Treatment for Agorioides

XML Treatment for Agorioides cherubino

XML Treatment for Agorioides papagena

XML Treatment for Agorioides

XML Treatment for Papuamyr

XML Treatment for Papuamyr omhifosga

XML Treatment for Papuamyr pandora
